# 含新药诱导化疗序贯自体造血干细胞移植、维持治疗策略治疗多发性骨髓瘤300例——单中心回顾性研究

**DOI:** 10.3760/cma.j.issn.0253-2727.2022.12.005

**Published:** 2022-12

**Authors:** 俊茹 刘, 景立 谷, 蓓晖 黄, 丽芬 邝, 美兰 陈, 外一 邹, 冬 郑, 荷花 王, 多荣 许, 娟 李

**Affiliations:** 中山大学附属第一医院，广州 510080 Department of Hematology, The First Affiliated Hospital, Sun Yat-sen University, Guangzhou, Guangdong 510080, China

**Keywords:** 多发性骨髓瘤, 自体造血干细胞移植, 微小残留病, Multiple myeloma, Autologous stem cell transplantation, Minimal residual disease

## Abstract

**目的:**

分析含新药诱导化疗序贯自体造血干细胞移植（auto-HSCT）、维持治疗策略治疗多发性骨髓瘤（MM）患者的预后及影响因素。

**方法:**

对近15年接受含新药方案诱导化疗序贯auto-HSCT、维持治疗的300例MM患者进行回顾性分析。

**结果:**

诱导化疗、auto-HSCT、维持治疗后的完全缓解（CR）率分别为35.3％、55.2％、72.4％，≥非常好的部分缓解（VGPR）率分别为80.0％、89.2％、93.4％；PAD方案（硼替佐米+脂质体阿霉素+地塞米松）诱导化疗的≥VGPR率、总反应率（ORR）均高于VD方案（硼替佐米+地塞米松），PAD方案和RAD方案（来那度胺+脂质体阿霉素+地塞米松）相比，CR率、≥VGPR率以及ORR差异均无统计学意义；诱导化疗、auto-HSCT、维持治疗后流式细胞术微小残留病（MRD）阴性患者在CR患者中的占比分别为18.8％（54例）、41.4％（109例）、58.7％（142例）。中位随访时间48.5（7.4～171.3）个月，全部MM患者中位疾病进展时间（TTP）为78.7个月，中位总生存（OS）时间为109.0个月；R-ISS Ⅰ、Ⅱ、Ⅲ期患者的中位TTP时间分别为111.8、77.4、30.6个月，中位OS时间分别为118.8、91.4、48.5个月。在治疗的不同阶段获得CR和MRD阴性患者的TTP、OS时间均较未获得CR和MRD阴性患者明显延长。在诱导化疗阶段获得CR的伴有高危细胞遗传学患者，其TTP短于不伴高危细胞遗传学患者；诱导化疗阶段获得MRD阴性的高危细胞遗传学患者，其TTP和不伴高危细胞遗传学患者相比差异无统计学意义。多因素分析显示，R-ISS分期在不同治疗阶段都是影响TTP和OS的不良预后因素；患者在接受治疗后，治疗获得的疗效就成为新的独立不良预后因素。

**结论:**

含新药诱导化疗序贯auto-HSCT、维持治疗的MM整体治疗策略具有较好的远期疗效，但高危MM患者获益不明显。

## Abstract (en)

**Objective:**

To examine the survival and influential factors of an integrated approach of novel agents, autologous hematopoietic stem cell（auto-HSCT）, and maintenance therapy in patients with multiple myeloma（MM）patients from a single center over the past 15 years.

**Methods:**

In our center, 300 MM patients who received an integrated strategy of new agents, auto-HSCT, and maintenance therapy over 15 years were retrospectively and prospectively analyzed.

**Results:**

The complete remission rates（CR）and ≥very good partial remission rates（VGPR）following induction therapy, transplantation, and maintenance therapy were respectively 35.3％ and 55.2％, 72.4％ and 80.0％, 89.2％, and 93.4％. When compared to patients receiving double-drug induction, the ≥VGPR and ORR of patients receiving triple-drug induction were improved. No difference existed in CR, ≥VGPR, and ORR between the PAD（bortezomib + liposome doxorubicin+ dexamethasone）and RAD（lenalidomide + liposome doxorubicin + dexamethasone）regimens, but the benefits speed differed. The negative rate of flow minimal residual disease following induction, transplantation, and maintenance was 18.8％（54 cases）, 41.4％（109 cases）, and 58.7％（142 cases）, respectively. The median time to progress（TTP）was 78.7 months and the median overall survival（OS）was 109 months. The median TTP for RISS-Ⅰ-Ⅲ patients were 111.8 months, 77.4 months, and 30.6 months, and the median OS was 118.8 months, 91.4 months, and 48.5 months, respectively. At various points during treatment, the TTP and OS of patients obtaining CR and MRD negative were longer than those of patients who did not obtain CR and MRD negative. TTP was noticeably shorter in high-risk cytogenetic patients compared to standard-risk patients even when CR was acquired during induction. There was no difference in TTP between patients with high-risk cytogenetics and those with standard-risk cytogenetics if MRD negative was acquired during induction. According to a multivariate analysis, the R-ISS stage was a poor predictor of TTP and OS at various treatment intervals. Therapeutic effectiveness was a newly independent prognostic factor following treatment.

**Conclusion:**

A median survival of almost 10 years is possible for MM patients who receive an integrated strategy of induction regimens followed by auto-HSCT and maintenance therapy, which significantly improves prognosis. However, this approach did not significantly benefit high-risk cytogenetic MM patients.

近年来，尽管新药层出不穷，自体造血干细胞移植（auto-HSCT）在多发性骨髓瘤（MM）治疗中依然具有着不可替代的重要地位，国内外多个指南均推荐新药序贯auto-HSCT作为年龄≤65岁MM患者的一线治疗选择[1]–[4]。在新药时代，虽然auto-HSCT的流程未发生明显改变，但移植的每个阶段如诱导化疗、造血干细胞动员、预处理、移植后维持治疗以及支持治疗均有一定进展[5]–[6]。本中心自2006年12月开始应用含新药方案诱导序贯auto-HSCT、维持治疗的整体策略治疗MM患者，其中100例和200例移植的相关结果已发表[7]–[8]，目前已有300例患者接受含新药诱导化疗序贯auto-HSCT、维持治疗的整体策略治疗，本研究对接受该策略治疗患者的远期疗效进行分析。

## 病例与方法

一、病例来源和研究方法

本研究为回顾性研究，纳入2006年12月至2021年4月期间在本中心接受含新药方案诱导化疗序贯auto-HSCT、维持治疗的300例MM患者。所有患者均符合《血液病诊断及治疗标准》（2014年前诊断病例）或IMWG 2014诊断标准（2014年以后诊断病例）。

二、治疗方案

1. 诱导化疗：所有患者均采用含新药硼替佐米和（或）来那度胺的诱导化疗方案：VD方案（硼替佐米+地塞米松）65例（21.7％），PAD方案（硼替佐米+脂质体阿霉素+地塞米松）209例（69.7％），RAD方案（来那度胺+脂质体阿霉素+地塞米松）17例（5.7％），VRD方案（硼替佐米+来那度胺+地塞米松）8例（2.6％），VCD方案（硼替佐米+环磷酰胺+地塞米松）1例（0.3％）。具体用药见文献[8]–[9]。中位诱导疗程数为4（1～10）疗程。

2. 造血干细胞动员和采集：275例患者采用大剂量环磷酰胺（CTX）+G-CSF方案进行外周血干细胞动员（其中84例患者应用长效G-CSF），4例患者应用G-CSF单药动员外周血干细胞。21例患者应用G-CSF单药动员后采集骨髓。

3. 预处理方案：美法仑（Mel）200 mg/m^2^方案136例（45.3％），Mel 140 mg/m^2^方案17例（5.7％），Mel 100 mg/m^2^方案3例（1.1％），接受硼替佐米联合Mel方案5例（1.7％），CVB方案139例（46.3％），具体用药方法见文献[7]。

4. 维持治疗：移植后血象恢复即开始给予维持治疗。早期患者维持治疗方案采用沙利度胺200 mg/d或重组人干扰素α2b注射液3×10^6^ U每周3次（半年后减为每周2次），或联合应用沙利度胺和重组人干扰素α2b注射液，不耐受或存在用药禁忌证者改为来那度胺10 mg/d维持。自2015年后，维持治疗逐渐改为来那度胺10 mg/d或VD方案、伊沙佐米。维持治疗持续到疾病进展，中位维持治疗时间为30.8（2.1～162.3）个月。

三、疗效评价

采用IMWG 2005疗效标准[10]评价疗效，分为完全缓解（CR）、非常好的部分缓解（VGPR）、部分缓解（PR）、疾病稳定（SD）和疾病进展（PD）。CR定义为血清和尿免疫固定电泳阴性、软组织浆细胞瘤消失、骨髓中浆细胞<5％。在诱导化疗结束后，auto-HSCT后第1年每3个月1次、1年以后每半年1次进行疗效评价，对达到CR的患者应用流式细胞术进行骨髓MRD评估[11]，抗体标志为CD38/CD45/CD19/CD20/CD56/CD54/CD138/cκ/cλ。检测1×10^6^个细胞，以检出≥20个异常表型的浆细胞为阳性，检测的敏感度为10^−4^～10^−5^。总体反应率（ORR）为CR率、VGPR率及PR率之和。

四、随访

采用查阅住院/门诊病例及电话随访方式获得患者生存情况。所有患者均随访至2021年7月31日，中位随访时间为48.5（7.4～171.3）个月。总生存（OS）时间指患者从接受诱导化疗至各种原因导致死亡或随访截止的时间，疾病进展时间（TTP）指从接受诱导化疗至疾病客观进展或随访截止的时间。

五、统计学处理

采用SPSS 13.0统计软件进行分析。计数资料比较采用*χ*^2^检验，计量资料采用*t*检验，不符合正态分布的计量资料采用秩和检验。生存分析采用Kaplan-Meier法进行分析，采用Cox回归进行影响生存的单因素和多因素分析，*P*<0.05为差异具有统计学意义。

## 结果

一、一般资料

全部300例MM患者中，男185例（61.7％），女115例（38.3％）；新诊断MM 277例（92.3％），复发/难治MM 23例（7.7％）；男185例（61.7％），女115例（38.3％）；中位发病年龄53（27～69）岁；M蛋白类型：IgG155例（51.7％），IgA 68例（22.7％），IgD 11例（3.7％），轻链型65例（21.6％），胞浆κ型1例（0.4％）；R-ISS分期：Ⅰ期72例（24.0％），Ⅱ期174例（58.0％），Ⅲ期37例（12.3％）；初诊297例有肌酐检测结果，≥177 µmol/L为43例（14.3％）；初诊HGB>100 g/L、≤100 g/L分别为130例（43.3％）、168例（56.0％），PLT>100×10^9^/L、≤100×10^9^/L分别为271例（90.3％）、16例（5.3％）；285例有LDH检测结果，≥240 U/L者45例（15.8％）；295例有血钙检测结果，≥2.75 mmol/L者36例（12.2％）；细胞遗传学分析结果如下：13q− 27.7％（83/235），1q21扩增27.0％（81/209），t（11;14）10.0％（21/211），t（4;14）19.4％（41/211），t（14;16）2.8％（6/211），17p− 7.8％（19/245），双打击2.9％［6/209，均为t（4;14）合并17p−］。

二、疗效评估

1. 传统疗效评估：诱导治疗后CR率、≥VGPR率分别为35.3％（106/300）、80.0％（240/300）。移植后CR率、≥VGPR率分别为55.2％（164/297）、89.2％（265/297），维持治疗后CR率、≥VGPR率分别为72.4％（197/272）、93.4％（254/272）。移植后与诱导治疗后相比，CR率、≥VGPR率差异均有统计学意义（*χ*^2^＝128.600，*P*<0.001；*χ*^2^＝9.745，*P*＝0.002）。

PAD诱导方案组诱导后的≥VGPR率、ORR率均高于VD诱导方案组，PAD方案和RAD方案组比较，CR率、≥VGPR率以及ORR率差异均无统计学意义（表1）。

**表1:** 不同诱导化疗方案多发性骨髓瘤患者诱导后疗效比较［例（％）］

组别	例数	CR	VGPR	PR	≥VGPR	ORR
VD方案	65	18（27.7）	23（35.4）	16（24.6）	41（63.1）	57（87.7）
PAD方案	209	76（36.4）	102（48.8）	23（11.0）	178（85.2）^a^	201（96.2）^a^
RAD方案	17	7（41.2）^b^	5（29.4）	4（23.5）	12（70.6）^b^	16（94.1）^b^

注 CR：完全缓解；VGPR：非常好的部分缓解；PR：部分缓解；ORR：总体反应率（≥PR率）；VD方案：硼替佐米+地塞米松；PAD方案：硼替佐米+脂质体阿霉素+地塞米松；RAD方案：来那度胺+脂质体阿霉素+地塞米松。与VD方案组比较，^a^*P*<0.05；与PAD方案组比较，^b^*P*>0.05

2. 流式细胞术MRD评估：在获得CR患者中进行流式细胞术MRD检测，未达到CR疗效者直接判定为MRD阳性，不进行MRD检测。诱导治疗后18.8％（54/287）的患者获MRD阴性，移植后3个月MRD阴性患者占比为41.4％（109/263），与诱导治疗后MRD阴性率相比差异有统计学意义（*χ*^2^＝83.08，*P*<0.001）。维持治疗后MRD阴性率达58.7％（142/242），与移植后3个月相比差异有统计学意义（*χ*^2^＝117.6，*P*<0.001）。

三、造血干细胞的动员和采集

275例患者接受大剂量CTX+G-CSF方案动员外周血造血干细胞，一次动员成功率为91.3％（251/275），84例应用CTX±长效G-CSF的患者中有80例（95.2％）一次动员成功。15例患者第二次动员CTX增至5 g/m^2^或应用G-CSF单药或联合普乐沙福后动员成功，5例患者一次动员失败后改为骨髓移植，3例患者一次动员失败后改为外周血+骨髓移植，1例患者两次动员失败后改为外周血+骨髓移植。采集单个核细胞（MNC）（6.57±3.66）×10^8^/kg，CD34^+^细胞为（6.20±5.57）×10^6^/kg。PAD诱导方案组获得CD34^+^细胞数高于RAD/VRD诱导方案组［（6.99±5.91）×10^6^/kg对（3.48±2.94）×10^6^/kg，*t*＝2.921，*P*＝0.031］。

四、移植模式和移植后造血重建

271例患者接受外周血造血干细胞移植，25例接受骨髓移植，4例接受外周血+骨髓移植。未发生移植相关死亡。中位粒细胞、血小板重建时间分别为10（6～43）d、12（1～180）d。PAD、RAD/VRD诱导方案组中位粒细胞重建时间分别为10（6～35）d、10（9～12）d（*z*＝−0.591，*P*＝0.208），中位血小板重建时间分别为11（6～180）d、12（10～18）d（*z*＝−1.202，*P*＝0.251）。美法仑预处理组和CVB方案预处理组中位粒细胞重建时间分别为11（6～43）d、10（7～35）d（*z*＝−7.307，*P*<0.001），中位血小板重建时间分别为13（1～120）d、10（6～180）d（*z*＝−6.750，*P*＝0.035）。

五、总生存

本组患者中位随访时间48.5（7.4～171.3）个月，中位TTP时间为78.7（95％*CI* 59.5～97.9）个月，中位OS时间为109.0（95％*CI* 86.8～131.2个月，OS曲线见图1。美法仑预处理组和CVB方案预处理组的中位TTP时间分别为79.9（95％ *CI* 48.8～111.0）、78.7个月（*χ*^2^＝0.094，*P*＝0.759），中位OS时间分别为111.3（95％*CI* 87.9～134.7）、92.0（95％*CI* 64.5～119.5）个月（*χ*^2^＝1.284，*P*＝0.257）。

**图1:**
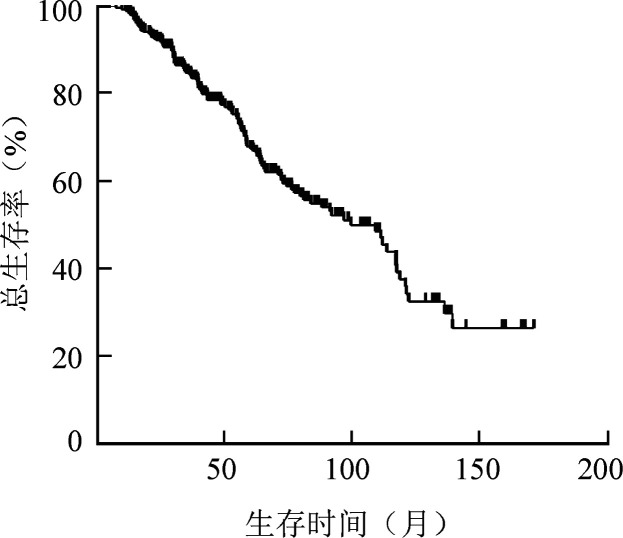
300例接受含新药方案诱导化疗序贯auto-HSCT、维持治疗多发性骨髓瘤患者的总生存曲线

六、影响TTP和OS的预后因素分析

1. R-ISS分期对TTP和OS的影响：R-ISS Ⅰ、Ⅱ、Ⅲ期患者的中位TTP时间分别为111.8、77.4（95％ *CI* 49.2～105.6）、30.6（95％ *CI* 25.7～35.5）个月（*χ*^2^＝18.86，*P*<0.001），三组患者的中位OS时间分别为118.8、91.4（95％ *CI* 64.7～118.1）、48.5（95％ *CI* 25.0～72.0）个月（*χ*^2^＝23.10，*P*<0.001）。

2. 高危细胞遗传学对TTP和OS的影响：伴有高危细胞遗传学［具备t（4；14）、t（14；16）、17p−其中之一者］患者的中位TTP和OS时间均明显低于不伴有高危细胞遗传学患者［TTP：88.2（95％ *CI* 77.2～99.1）个月对42.17（95％ *CI* 25.6～58.7）个月，*χ*^2^＝15.008，*P*<0.001；OS：117.3（95％ *CI* 81.2～153.4）个月对96.8（95％ *CI* 48.9～144.6）个月，*χ*^2^＝5.647，*P*＝0.017］。伴有t（4；14）、17p−表达患者的TTP时间分别为47.8（95％*CI* 28.1～67.5）、24.8（95％*CI* 14.1～35.4）个月，均低于不伴相应细胞遗传学异常［87.8（95％*CI* 77.1～98.2）个月］（*χ*^2^＝6.310，*P*＝0.012；*χ*^2^＝13.102，*P*<0.001）；伴17p−患者的OS时间明显低于不伴17p−的患者［48.5（95％*CI* 0～100.7）个月对99.5（95％*CI* 70.9～128.2）个月，*χ*^2^＝5.581，*P*＝0.018］。

3. 不同治疗阶段疗效对TTP和OS的影响：诱导化疗后获得、未获得CR患者的中位TTP时间分别为111.8（95％*CI* 81.6～142.0）、53.5（95％*CI* 36.1～70.9）个月（*χ*^2^＝6.589，*P*＝0.010）；移植后获得、未获得CR患者的中位TTP时间分别为91.4（95％*CI* 78.4～104.3）、47.8（95％*CI* 39.2～56.4）个月（*χ*^2^＝10.023，*P*＝0.002）；维持后获得、未获得CR患者的中位TTP时间分别为87.8（95％*CI* 72.4～103.1）、34.9（95％*CI* 20.4～49.4）个月（*χ*^2^＝18.812，*P*<0.001）；诱导后获得、未获得CR患者的中位OS时间分别为117.3（95％*CI* 97.7～136.9）、83.8（95％*CI* 57.9～109.7）个月（*χ*^2^＝4.179，*P*＝0.042），移植后获得、未获得CR患者的中位OS时间分别为117.5（95％*CI* 107.2～127.8）、74.4（95％*CI* 52.1～96.7）个月（*χ*^2^＝12.695，*P*<0.001），维持治疗后获得、未获得CR患者的中位OS时间分别为118.8（95％*CI* 100.1～137.4）、72.8（95％*CI* 41.3～104.3）个月（*χ*^2^＝15.253，*P*<0.001），差异均有统计学意义。

诱导后获得（54例）、未获得MRD阴性患者（233例）的中位TTP时间分别为未达到、57.9（95％*CI* 38.0～77.9）个月（*χ*^2^＝10.09，*P*＝0.001），移植后获得（109例）、未获得MRD阴性（154例）患者的中位TTP时间分别为99.9、47.2（95％*CI* 39.9～54.5）个月（*χ*^2^＝16.417，*P*<0.001），维持后获得（142例）、未获得（100例）MRD阴性患者的中位TTP时间分别为未达到、34.9（95％*CI* 23.3～46.4）个月（*χ*^2^＝35.060，*P*<0.001）。诱导后获得、未获得MRD阴性患者的中位OS时间分别为未达到、91.4（95％*CI* 62.8～119.9）个月（*χ*^2^＝4.085，*P*＝0.048），移植后获得、未获得MRD阴性患者的中位OS时间分别为未达到、74.4（95％*CI* 60.5～88.3）个月（*χ*^2^＝8.752，*P*＝0.003），维持后获得、未获得MRD阴性患者的中位OS时间分别为未达到、72.8（95％*CI* 58.6～87.0）个月（*χ*^2^＝13.499，*P*<0.001）。

4. 不同阶段获得CR和MRD阴性对伴有高危细胞遗传学患者TTP的影响：在诱导后获得CR患者中，伴有（17例）、不伴有（60例）高危细胞遗传学患者的中位TTP时间分别为37.9（95％*CI* 23.7～52.2）个月、未达到（*χ*^2^＝7.664，*P*＝0.006）。在移植后获得CR的患者中，伴有（28例）、不伴有（91例）高危细胞遗传学患者的中位TTP时间分别为42.2（95％*CI* 17.0～67.3）个月、未达到（*χ*^2^＝10.166，*P*＝0.001）。在维持治疗后获得CR患者中，伴有（32例）、不伴有（107例）高危细胞遗传学患者的中位TTP时间分别为47.8（95％*CI* 33.9～61.8）个月、91.4（95％*CI* 80.2～102.5）个月（*χ*^2^＝9.616，*P*＝0.002）。

在诱导化疗后MRD阴性患者中，伴有（9例）、不伴有（35例）高危细胞遗传学患者的中位TTP时间分别为37.9个月、未达到（*χ*^2^＝2.762，*P*＝0.097）；在移植后MRD阴性患者中，伴有（19例）、不伴有（68例）高危细胞遗传学患者的中位TTP时间分别为52.8（95％*CI* 30.6～75.0）、99.9（95％*CI* 85.3～114.5）个月（*χ*^2^＝4.909，*P*＝0.027）；在维持治疗后MRD阴性患者中，伴有（25例）、不伴有（87例）高危细胞遗传学患者的中位TTP时间分别为79.9（95％*CI* 36.5～123.3）、99.9（95％*CI* 82.9～116.9）个月（*χ*^2^＝5.118，*P*＝0.024）。

5. 影响TTP和OS的多因素分析：多因素分析表明，无论在什么阶段，R-ISS分期都是TTP和OS的不良预后因素。患者在接受治疗后，治疗的疗效就成为新的独立不良预后因素，无论是诱导化疗、移植以及维持治疗阶段不能获得CR都是影响TTP和OS的独立危险因素（表2）。

**表2:** 影响接受含新药方案诱导化疗序贯auto-HSCT、维持治疗的300例多发性骨髓瘤（MM）患者疾病进展（TTP）时间和总生存（OS）时间的多因素分析

变量	TTP		OS	
	*HR*（95% *CI*）	*P*值	*HR*（95% *CI*）	*P*值
初诊				
R-ISS Ⅱ/Ⅲ	2.757（1.688~4.502）	<0.001	2.873（1.288~3.395）	0.003
诱导化疗后				
R-ISS Ⅱ/Ⅲ	2.357（1.485~3.742）	<0.001	2.605（1.613~4.209）	<0.001
未达CR	1.588（1.057~2.386）	0.026	1.602（1.017~2.525）	0.042
auto-HSCT后				
R-ISS Ⅱ/Ⅲ	2.540（1.596~4.041）	<0.001	2.916（1.803~4.716）	<0.001
未达CR	1.759（1.220~2.536）	0.002	2.026（1.355~3.029）	0.001
维持治疗后				
R-ISS Ⅱ/Ⅲ期	2.647（1.660~4.220）	<0.001	2.810（1.699~4.648）	<0.001
未达CR	2.189（1.475~3.249）	<0.001	2.124（1.372~3.288）	0.001

注 R-ISS：修订的国际分期系统；CR：完全缓解

## 讨论

本中心自2006年起应用含新药方案诱导化疗序贯auto-HSCT、维持治疗的整体策略治疗MM患者，迄今已15年，结果显示该治疗策略可使MM患者获得的中位OS时间为109个月，中位TTP时间为78.7个月，接近美国梅奥中心报道的数据[12]。因此，从总体来说，含新药序贯auto-HSCT可以明显延长MM患者的长期生存，也进一步证实了auto-HSCT的重要地位。

对不同治疗阶段获得的疗效进行分析，患者在接受auto-HSCT和维持治疗后的CR率、MRD阴性率均得到明显的提高，这归结于采用大剂量细胞毒药物进行后续造血干细胞动员及预处理。有别于诱导治疗应用的蛋白酶体抑制剂，维持治疗大多为免疫调节剂，作用机制亦不相同，因此患者可进一步通过auto-HSCT和维持治疗获益。对不同诱导方案的疗效进行分析，三药诱导的疗效明显优于两药诱导，含来那度胺的三药诱导和含硼替佐米的三药诱导方案近期总的疗效相比并无差异，但RAD方案诱导起效较为缓慢，两疗程的CR率明显低于PAD方案[9]。

目前MM动员方案主要包括G-CSF单药或联合普乐沙福、大剂量化疗联合G-CSF[13]–[15]。本研究大多数病例采用大剂量CTX+G-CSF动员，个别患者因考虑可能不能耐受CTX而采用G-CSF单药。对含来那度胺诱导治疗的患者干细胞采集数量明显低于含硼替佐米方案，我们之前的研究显示RAD化疗超过3疗程即会明显影响干细胞采集[9]。目前我们也正在开展对个别动员疗效欠佳患者采用按需使用普乐沙福的方案，以期降低动员失败率。按需给药方案在提高动员效果的同时也降低了移植成本[16]。

诱导治疗缓解程度越深，移植后的PFS期和OS时间越长，尤其是获得MRD阴性的患者[17]。本组病例中，无论在诱导化疗后、移植后还是维持治疗后，只要能获得CR和MRD阴性，均较未获得相应疗效患者的TTP和OS要好。但对于伴有高危细胞遗传学的患者，即使在诱导阶段获得CR，其TTP仍较不伴高危细胞遗传学患者明显缩短。进一步分析显示，如果在诱导阶段获得MRD阴性，则伴有高危细胞遗传学患者的TTP和不伴高危细胞遗传学患者相比无统计学差异。因此，对于MM患者来说，无论在哪个治疗阶段，均应尽可能获得CR[18]。而对于伴有高危细胞遗传学的患者，应将诱导化疗后获得MRD阴性作为目标[19]。

虽然MM患者的远期疗效通过整体治疗体系明显提高，但对于高危患者来说仍未能达到理想效果，R-ISS Ⅲ期患者中位TTP、OS时间仅为30.6、48.5个月，高危患者的治疗需求仍未满足。目前专门针对高危患者的治疗探索也正在开展，德国GMMG-CONCEPT研究应用CD38单抗Isatuximab联合KRD方案（卡非佐米+来那度胺+地塞米松）用于高危NDMM患者一线治疗，可达迅速且深度的缓解，61％患者达到MRD阴性[20]。英国Optimism研究应用Dara-VRCD诱导治疗高危MM，也都取得很好的效果，这些研究在诱导治疗都加入了单克隆抗体，使得高危患者在诱导治疗阶段就获得良好的MRD转阴率，以此来克服高危细胞遗传学的不良预后[21]。FORTE研究应用卡非佐米和来那度胺的联合方案进行维持治疗，结果显示可改善伴高危细胞遗传学患者的不良预后[22]。

MM患者的预后因素尚未达成共识。以往研究大多只包含患者诊断时的因素，并未纳入治疗相关因素[23]–[25]。本研究对影响长期生存的危险因素进行分析，发现无论在哪一个治疗阶段，R-ISS分期都是影响TTP和OS的不良预后因素。患者在接受治疗后，前一治疗阶段的疗效就成为新的独立不良预后因素，无论是诱导化疗、移植以及维持治疗后不能获得CR都是影响TTP和OS的独立危险因素。

美国MD Anderson肿瘤中心在2020年ASH会议上报道了近25年来接受auto-HSCT患者的生存情况[26]，2012–2015年期间患者的生存期优于1990–2001年期间患者，考虑与新药的应用以及auto-HSCT各个阶段的优化相关。梅奥中心也得出类似的结果[12]，按诊断时间将患者分为2004–2008、2009–2013和2014–2018，中位生存期随时间段逐渐延长。本组病例中位生存期达109个月，较既往100、200例患者报道的数据有所延长，不同时间段生存的改善归结于每个阶段治疗手段的改进和支持治疗的进展，包括：①诱导治疗由二药联合改为三药联合；②干细胞动员方案由大剂量G-CSF+短效G-CSF改为联合长效G-CSF提高动员成功率；③维持治疗由沙利度胺、甘乐能调整为来那度胺、含硼替佐米等方案；④支持治疗例如血小板受体激动剂的应用可部分克服移植后巨核重建障碍的问题。这也进一步说明，新药和auto-HSCT的进一步优化仍是我们的研究方向。

综上所述，本中心应用含新药诱导化疗序贯auto-HSCT、维持治疗这一整体治疗策略治疗MM患者，显示了较好的远期疗效，但高危患者通过本策略获益并不明显。相信随着各阶段治疗方案的优化，终将克服高危患者的不良预后。
